# Bandwidth Extension in a Mid-Link Optical Phase Conjugation

**DOI:** 10.3390/s22176385

**Published:** 2022-08-24

**Authors:** Paweł Rosa, Giuseppe Rizzelli Martella, Mingming Tan

**Affiliations:** 1 National Institute of Telecommunications, Szachowa 1, 04-894 Warsaw, Poland; 2 LINKS Foundation, Via Piercarlo Boggio 61, 10138 Torino, Italy; 3 Aston Institute of Photonics Technologies, Aston University, Birmingham B4 7ET, UK

**Keywords:** Raman amplification, optical fiber communications, optical phase conjugation

## Abstract

In this paper, we investigate various designs of distributed Raman amplifier (DRA) to extend amplification bandwidth in mid-link optical phase conjugation (OPC) systems and compare bands 191–197 THz and 192–198 THz giving a total bandwidth of 6 THz using a single wavelength pump. We demonstrate the use of highly reflective fiber Bragg grating (FBG) to minimize gain variation across a WDM grid by optimizing forward and backward pump powers as well as the wavelength of FBGs for original and conjugated channels. Finally, we also simulate OSNR and Kerr nonlinear reduction as a product of signals asymmetry and nonlinear phase shift (NPS) for all channels.

## 1. Introduction

Optical phase conjugator (OPC) in a long-haul transmission system can effectively compensate for both linear (e.g., chromatic dispersion) and nonlinear (e.g., the fiber Kerr nonlinearity) impairments, and therefore can improve the data capacity or transmission distance [1,2,3,4,5,6,7,8,9,10,11,12,13,14,15,16,17,18,19,20,21,22,23,24,25,26,27]. The efficiency of how much the fiber nonlinearity can be compensated assisted by mid-link OPC was limited by several factors, such as the slope of the chromatic dispersion map of the transmission fiber and the signal power profile along the fiber [1,2,3,4,5,6]. The symmetry of chromatic dispersion slope can be tailored by optimizing the dispersion map using a combination of transmission fiber and dispersion compensating fiber [9]. This can be used together with erbium-doped fiber amplifiers (EDFA) which is the most widely used amplification technique; however, it requires special transmission fiber and dispersion compensating fiber to maintain the dispersion map symmetry. Another way to maximize fiber nonlinearity compensation efficiency with OPC is to use distributed Raman amplification to make the signal power profile symmetrical. Distributed Raman amplification generates optical gain using Raman pump lasers over the standard transmission fiber, providing distributed amplification along the whole fiber rather than a discrete or lumped amplification within a few meters of doped fiber as in EDFA. DRA can be highly flexible to specifically tailor the signal power profiles to be highly symmetrical before and after the OPC [3,4,5,6]. The pump wavelength can be adjusted with fiber Bragg gratings (FBG) with selected center wavelength [28,29,30,31,32,33,34,35,36,37,38,39,40]. The use of distributed Raman amplification can improve the maximum transmission distance or data capacity without the mid-link OPC (or the first half of the link before the OPC) and therefore if the symmetry of the link can be maintained at a very high level for all channels, the overall transmission performances or data capacity can be significantly improved using mid-link OPC due to the efficient compensation of fiber nonlinearity [4,5,6].

We show, for the first time, that the transmission bandwidth can be extended using FBGs at two different wavelengths for transmitted and conjugated channels in mid-link optical phase conjugation. The novel design approach allows for the highest OPC bandwidth using Raman amplification and gives the lowest asymmetry for a single channel up to date for designs of distributed Raman amplification schemes which are aimed to improve the symmetry of the link for the transmission systems.

## 2. Distributed Raman Amplification

In telecommunication systems, there are several Raman configurations that can be used. In a single span unrepeatered submarine transmission, second order bi-directional pumping with two FBGs in the front and the end of the span provides great signal power distribution along the fiber compared to first order amplification, which ultimately increases transmission reach [37,38,39,40]. Depending on the application, the same approach might not work in long-haul transmission. Forward pumping can help with signal power distribution creating a quasi-lossless transmission medium [32]; however, the benefits of low noise and signal power variation become meaningless in coherent data transmission due to forward relative intensity noise (RIN) [32,33,34,35,36]. In [36] we compared six different Raman configurations, including first order, second order and dual order and have shown, experimentally, that bi-directionally distributed Raman amplification with a single FBG at the end of the transmission span, forming a half-open cavity with random distributed feedback (DFB) lasing [28], significantly decreases accumulation of amplified spontaneous emission (ASE) noise built up across the transmission span and keeps signal power variation low [28,29,30,31,32,33,34,35,36,37,38,39,40], extending data transmission by almost 900 km to a record distance of 7915 km. In this particular scheme the forward Raman laser pump at 1366 nm amplifies the backward propagating random DFB lasing at the frequency specified by the wavelength of the FBG. This approach allows for a RIN transfer reduction [32,33,34,35,36] from the noisy forward Raman pump to the Stokes-shifted light, becoming an efficient solution for a long-haul coherent data transmission format [33,34,35,36].

**Figure 1:**
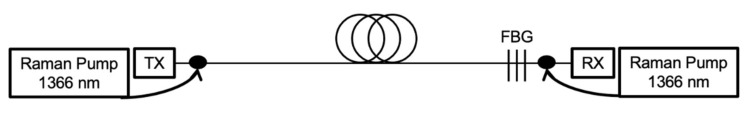
Raman fiber laser-based amplifier with a half-open cavity random lasing.

**Figure 2:**
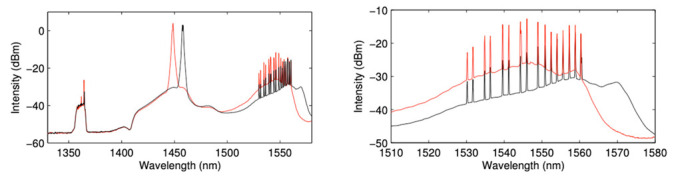
The Raman gain shift using FBGs centered at 1448 nm (red) and 1458 nm (black). The right figure is zoomed on the 16 WDM signals wavelength range.

The schematic design of the random DFB Raman laser amplifier is shown in Figure 1. Two Raman fiber laser pumps downshifted in wavelength to 1366 nm (approximately two Stokes shifts from the signal) were located at each end of the standard single mode fiber (SMF). The span length was 60 km. A half-open cavity random DFB laser was formed at the wavelength of the FBG at the end of the fiber that amplifies original and conjugated WDM channels in the OPC system. We assume that fiber used for the FBGs is the same as transmission fiber, which is standard SMF fiber. By optimizing the wavelength of the FBG, rather than deploying a seed at different wavelength, the spectral gain profile of the amplified WDM signals can be modified and enhanced. This is visualized by an example shown in Figure 2 where we compare the Raman gain shift resulting from FBGs at different wavelength. To avoid polarization gain dependance in WDM transmission, Raman pump lasers at both ends and lasing at the wavelength of the FBG were fully depolarized.

## 3. Simulation

The spectral components of the OPC system described in Section 2 was simulated using an experimentally confirmed model [32,33,34,35,36,37,38,39,40] that takes into account residual Raman gain from the primary pump at 1366 nm to the signal in the C-band as well as pump depletion from both pumps to the lower order pumps and the signal components, double Rayleigh scattering (DRS) and amplified spontaneous emission (ASE) noise for each of the signals. Noise was calculated in a room temperature of 24 °C and 0.1 nm bandwidth. Wavelength dependent Raman gain coefficient and attenuation factor were independently chosen for each WDM component as well as primary pump and lasing at the frequency of the FBG using tables for the SMF. Rayleigh backscattering coefficients for Raman pump, lasing and signal were 1.0 × 10^−4^, 6.5 × 10^−5^ and 4.5 × 10^−5^ km^−1^, respectively. In [28] we showed that higher reflectivity (99%) provides lower nonlinear phase shift compared with 70% and 50% as well as better power efficiency performance without sacrificing output OSNR. To find the best gain profile, the center wavelength of high reflectivity (99%) FBGs was varied from 1456 to 1464 nm for WDM section of transmitted channels and from 1442 to 1454 nm for the conjugated copy with a 2 nm step. The loss and bandwidth of the FBG in the simulations was set to 0.2 dB and 200 GHz, respectively.

**Figure 3:**
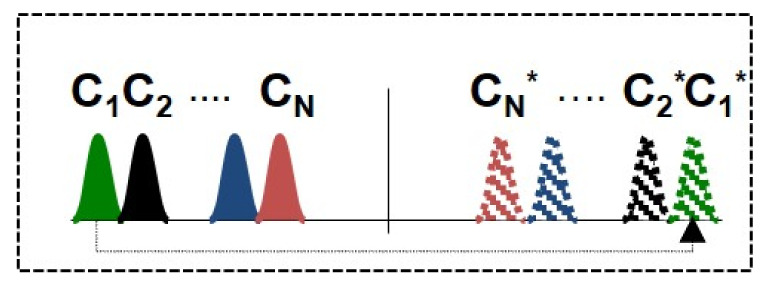
WDM grid of the transmitted (**left**) and the conjugated (**right**) WDM channels in a mid-link OPC system. “*” denotes conjugated channels.

Figure 3 illustrates the WDM grid of the mid-link OPC system. Total bandwidth of 6 THz consists of 30 transmitted WDM channels with 100 GHz spacing ranging from 192–194.9 THz and 30 WDM conjugated copies of the original signal in the range 195.1–198 THz (we also present results for 191–197 THz grid). We assumed 200 GHz as the guardband in the middle of the grid centered at 195 THz for the optical phase conjugator. The signal power profile of each WDM component of the original and conjugated signal was simulated independently.

Forward pump power of the Raman fibre laser at 1366 nm was simulated from 0.7 to 1.6 W with 100 mW step. Backward pump power was simulated to give 0 dB net gain for the channel under investigation, then the rest of the WDM channels were simulated with the same forward and backward pump powers: one set of results consists of 30 possible combinations (optimisation towards first channel CH1, second channel CH2 and so on). Conjugated signals were simulated with the same pocedure. To summarise, we simulated every possible combination varying forward pump power (0.7–1.6 W) for each installed FBG (1442–1454 nm for original and 1456–1464 nm for conjugated WDM grid) and finally compared asymmetry between both WDM channel sets to achieve the best overall asymmetry performance in an OPC system with second order Raman amplification.

The asymmetry was calculated using formula:

(1) $$\frac{\int_{0}^{L}\left|P_{1}\left(z\right)-P_{2}\left(L-z\right)\right|dz}{\int_{0}^{L}[P_{1}\left(z\right)+P_{2}\left(z\right)]/2dz}\times 100$$

where *L* is the span length and *P*_1_ and *P*_2_ represent signal power evolution of the transmitted and conjugated channels, respectively.

## 4. Results and Discussion

### 4.1. 191–197 THz WDM Grid

For a given WDM grid bandwidth, the key factors that need to be optimized for the best performance overall in a mid-link OPC system are the wavelength of the FBG and the forward and backward pump powers. Both optimizations must be done separately for the original and conjugated WDM grid. Our initial investigation was done for a WDM grid 191–197 THz. Firstly, we had to select the right set of FBGs that will have a performance impact for residual channels located at the beginning and the end of the band.

**Figure 4:**
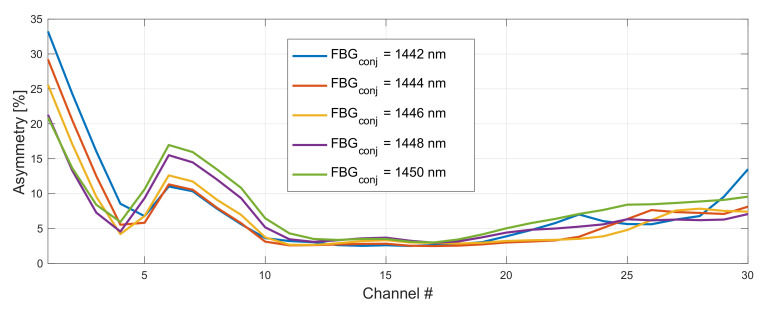
Impact of a FBG choice on asymmetry performance in 191–197 THz grid. Wavelength of a FBG for original WDM grid was set to 1462 nm and FBG for the conjugated copy was varied from 1442–1450 nm.

In Figure 4 we show the impact of the FBG wavelength on asymmetry performance in WDM grid under investigation. Here, the central wavelength of the FBG for original channels was set to 1462 nm (the best configuration) and the one of the FBG for conjugated channels was varied from 1442–1450 nm. The asymmetry of residual side channels can be improved by 12% (CH1: 21% with 1450 nm and 33% with 1442 nm). However, this advantage is lost for CH6 and CH7 where the asymmetry performance is reversed, hence we need average overall performance. In this case the matching FBGs pair was 1462 nm for original and 1446 nm for conjugated WDM grid (we also simulated FBG for original WDM grid from 1456–1464 nm, however, for clarity we only show the best performing configuration with 1462 nm). Next, we optimized forward and backward pump powers for original and conjugated WDM grid.

**Figure 5:**
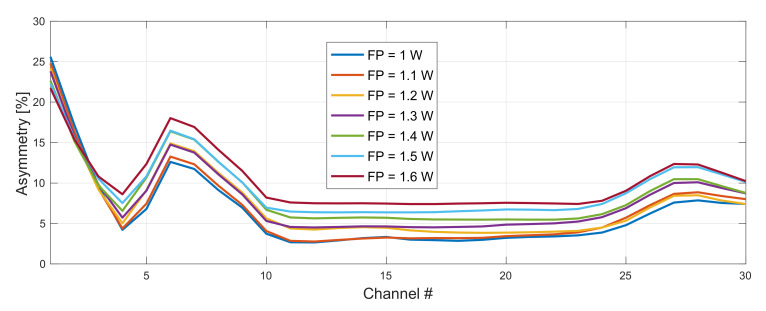
Impact of pump power choice (original WDM grid) on asymmetry performance. Forward pump power of the conjugated grid fixed to be 1.3 W. FBG for original and conjugated WDM grid was set to 1462 and 1446 nm, respectively.

In Figure 5 we show the impact of pump power on asymmetry performance for original and conjugated WDM grid. Forward pump power for both configurations was varied from 1–1.6 W. Backward pump power from 2.6–2.9 W for original (191–193.9 THz) and 1.6–1.9 W for conjugated (194.1–197 THz) WDM grid. The difference in backward pump power for each WDM grid results from wavelength dependent Raman gain and fiber attenuation.

By analyzing all possible combinations of FBG, forward and backward pump powers for original and conjugated WDM grid, we found the profile that gave the best average asymmetry of 6.4%; however, this figure is biased by first residual channels that are on the edge of Raman gain profile.

**Figure 6:**
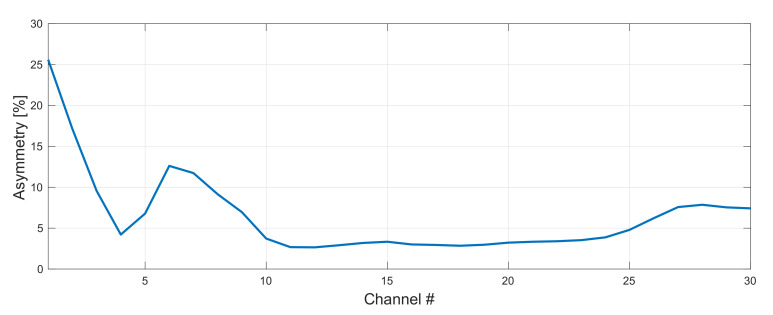
WDM grid with the best average asymmetry of 6.4%. Forward pump power was set to be 1 W and 1.3 W and FBG to 1462 nm and 1446 nm for original and conjugated WDM grid, respectively.

In Figure 6 we show the best profile with the following settings: FBG 1462 nm with forward pump power 1 W for the original, and FBG 1446 nm with 1.3 W forward pump power for the conjugated WDM grid.

### 4.2. 192–198 THz WDM Grid

Based on the results shown in Section 4.1, we decided to repeat the simulations with a grid shifted by 1 THz to avoid the worst performing channels in the beginning of the spectrum. The new grid was set to 192–198 THz. Based on the knowledge from the previous configuration we only tested the FBG sets 1458–1460 nm and 1450–1454 nm for original and conjugated WDM grid, respectively.

**Figure 7:**
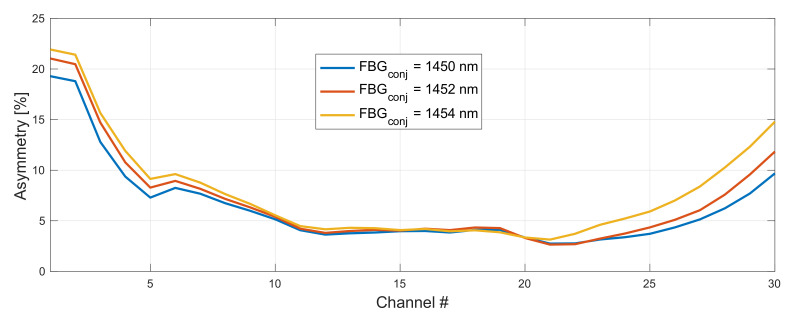
Impact of a FBG choice on asymmetry performance in 192–198 THz grid. FBG for original WDM grid was set to 1458 nm and conjugated varied from 1450–1454 nm.

**Figure 8:**
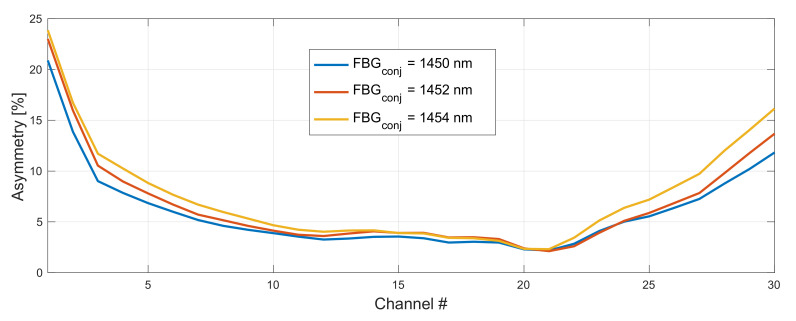
Impact of a FBG choice on asymmetry performance in 192–198 THz grid. FBG for original WDM grid was set to 1460 nm and conjugated varied from 1450–1454 nm.

**Figure 9:**
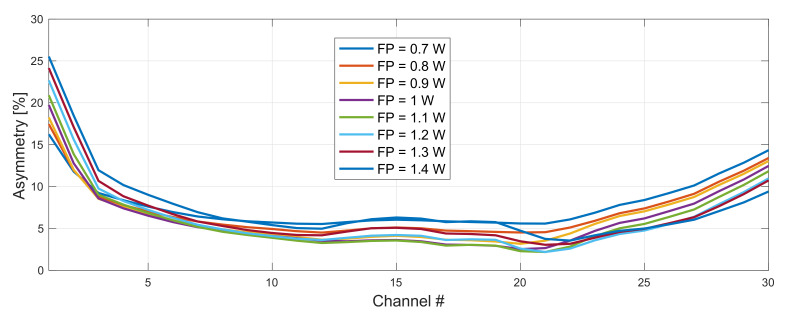
Impact of pump power choice (original WDM grid) on asymmetry performance. Forward pump power of the conjugated grid was fixed to be 1.3 W. FBG for original and conjugated WDM grid was set to 1460 and 1450 nm, respectively.

**Figure 10:**
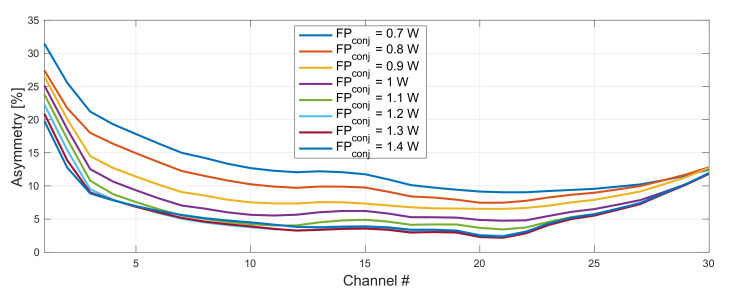
Impact of pump power choice (conjugated WDM grid) on asymmetry performance. Forward pump power of the original grid was fixed to be 1.1 W. FBG for original and conjugated WDM grid was set to 1460 and 1450 nm, respectively.

In Figure 7 and Figure 8, we show the impact of a FBG choice on asymmetry performance in 192–198 THz grid. The optimum configuration giving the lowest average asymmetry was with FBG set to 1460 nm and 1450 nm for the original (Figure 9) and conjugated (Figure 10) WDM grid, respectively. The right choice of a FBG helps to optimize the gain profile of the WDM band, which will particularly have an impact for an on–off gain of residual front and end channels. This will directly affect asymmetry performance.

After choosing the right FBG set for our WDM band we optimized forward and backward pump powers. This time we extended the range and simulated 0.7–1.4 W forward pump power. Backward pump power was stable for all possible configurations and oscillated within the 50 mW range around 2.3 W for the original and 1.8 W for the conjugated WDM grid.

**Figure 11:**
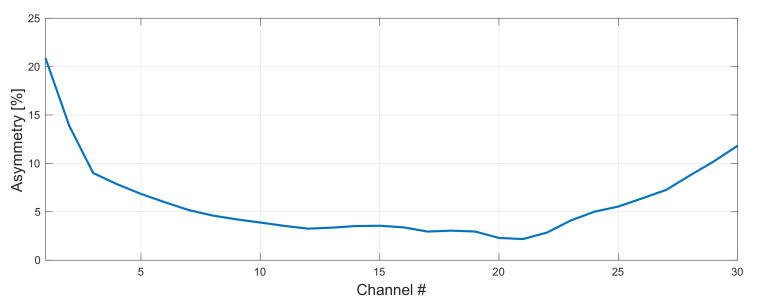
WDM grid with the best average asymmetry of 5.9%. Forward pump power was set to be 1.1 W and 1.3 W and FBG to 1460 nm and 1450 nm for original and conjugated WDM grid, respectively.

By shifting our band by 1 THz we managed to improve average asymmetry by 0.5% achieving 5.9% with only three channels (CH1, CH2 and CH30) with figures above 10%. In this regime (asymmetry below 10%) we exceeded 35 nm bandwidth of the C band (1530–1565 nm) from ~4.3 THz to 5.4 THz (27 WDM channels with asymmetry below 10%, giving total 54 channels). As seen in Figure 11, the majority of channels (CH7–CH24) were below 5%, which is a very impressive result covering 3.4 THz.

**Figure 12:**
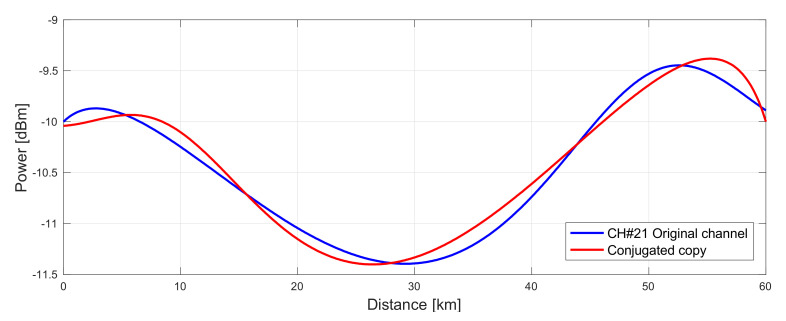
Signal power profile of original (blue) and conjugated (red) channel with lowest asymmetry of 2.1% in a 60 km standard SMF span.

In a single channel regime CH21 achieved asymmetry 2.1%, which is the lowest asymmetry in a 60 km span up to date (see Figure 12). This value most likely would be even lower if a single channel only would be simulated with the same configuration due to the Raman pump depletion. Signal power variation in the original and conjugated channels is very low, below 2 dB, comparing to 12 dB when using EDFA.

**Figure 13:**
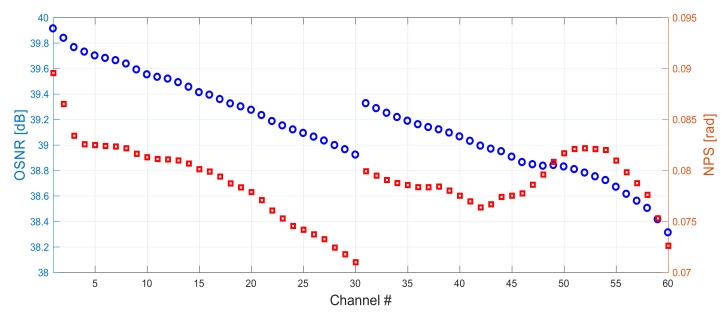
OSNR (blue) and NPS (red) simulations for original (CH1–CH30) and conjugated (CH31–CH60) channels.

In Figure 13 we show OSNR and NPS results for an optimized 6 THz WDM grid (192–198 THz) for both original (CH1–CH30) and conjugated (CH31–CH60) channels in a 60 km span. We can notice small OSNR variation within 1 dB range between the best and the worst performing channels for both simulated grids, thanks to the combined gain from the first order pump at 1366 nm and lasing at the wavelength of the FBG. NPS variation was also very low across all but the first two channels; however, at these values of NPS it is insignificant.

## 5. Conclusions

We present, for the first time, the use of half-open cavity random DFB Raman laser amplification with combinations of different FBGs to extend the bandwidth of the mid-link OPC system beyond the standard C band. We performed gain profile optimization in a 60 km standard SMF span utilizing a 6 THz bandwidth with total of 60 WDM channels. With a fine optimization of FBGs and pump powers, we achieved for the first time a record 5.9% average asymmetry in a 192–198 THz band using single wavelength 1366 nm Raman laser pump only in an OPC system. We also achieved 2.1% asymmetry performance for a single channel, which is the lowest asymmetry achieved to date in a 60 km standard SMF span length. The results show the potential for further improvement by utilizing 6 THz band using DWDM with a 25 GHz or even 12.5 GHz spacing that would increase the total transmission capacity of an OPC system by an order of magnitude.

## Data Availability

Original data is available at Aston Research Explorer (https://doi.org/10.17036/researchdata.aston.ac.uk.00000575).
